# The First Complete Genome Sequence of the Class *Fimbriimonadia* in the Phylum *Armatimonadetes*

**DOI:** 10.1371/journal.pone.0100794

**Published:** 2014-06-26

**Authors:** Zi-Ye Hu, Yue-Zhu Wang, Wan-Taek Im, Sheng-Yue Wang, Guo-Ping Zhao, Hua-Jun Zheng, Zhe-Xue Quan

**Affiliations:** 1 Department of Microbiology and Microbial Engineering, School of Life Sciences, Fudan University, Shanghai, China; 2 Shanghai-MOST Key Laboratory of Health and Disease Genomics, Chinese National Human Genome Center at Shanghai, Shanghai, China; 3 Department of Biotechnology, Hankyoung National Univeristy, Kyonggi-do, Republic of Korea; 4 Laboratory of Medical Foods, Shanghai Institute of Planned Parenthood Research, Shanghai, China; University of Florida, United States of America

## Abstract

In this study, we present the complete genome of *Fimbriimonas ginsengisoli* Gsoil 348^T^ belonging to the class *Fimbriimonadia* of the phylum *Armatimonadetes*, formerly called as candidate phylum OP10. The complete genome contains a single circular chromosome of 5.23 Mb including a 45.5 kb prophage. Of the 4820 open reading frames (ORFs), 3,000 (62.2%) genes could be classified into Clusters of Orthologous Groups (COG) families. With the split of rRNA genes, strain Gsoil 348^T^ had no typical 16S-23S-5S ribosomal RNA operon. In this genome, the GC skew inversion which was usually observed in archaea was found. The predicted gene functions suggest that the organism lacks the ability to synthesize histidine, and the TCA cycle is incomplete. Phylogenetic analyses based on ribosomal proteins indicated that strain Gsoil 348^T^ represents a deeply branching lineage of sufficient divergence with other phyla, but also strongly involved in superphylum *Terrabacteria*.

## Introduction

More than two decades ago, 12 phylum-level phylogenetic clusters have been added to the tree of life after the famous culture-independent survey done by the group of Norman Pace in Obsidian Pool, Yellowstone National Park [1]. These clusters, nominated OP1 to OP12, were unaffiliated to any cultured bacteria at the time, and were therefore described as candidate phyla.

Among the 12 proposed bacterial candidate phyla found in Obsidian Pool, some have earned their official membership in the domain *Bacteria* due to the cultured strains have been obtained, including candidate phylum OP10, which has been proposed as phylum *Armatimonadetes* according to the mesophilic isolate strain YO-36^T^ [2]. Phylum *Armatimonadetes* has three characteristics: i), relatively high abundance. In ARB-Silva reference database, 1/1000 sequences were classified as candidate phylum OP10 [3]. ii), various preferred habitats. Members of this bacterial phylum have been detected in numerous 16S rRNA gene surveys from environments that vary greatly in physical and biogeochemical characteristics [3]–[6]. iii), difficult to obtain pure culture. Since discovery, besides strain YO-36^T^, only strains T49^T^ and P488 classified to class *Chthonomonadetes* [5] and strain Gsoil 348^T^ classified to class *Fimbriimonadia* have been reported [4].

Here, we present the completed genome sequence of strain Gsoil 348^T^. Strain Gsoil 348^T^ was isolated from soil of ginseng field by selecting micro-colonies from an oligotrophic agar medium after two months incubation [4]. Strain Gsoil 348^T^ is mesophilic, strictly aerobic, non-motile and rod-shaped, and cannot utilize most of substrates as sole carbon sources and only grows in low nutrient media [4]. We proposed novel class *Fimbriimonadia* based on strain Gsoil 348^T^ [4]. The class specific primer pair was used for diversity and abundance analyses and we found that bacteria belonging to class *Fimbriimonadia* are spread across various environments such as rhizosphere, hypersaline water, anaerobic sludge, marine sediment and hotspring water, in the range of 0.01% to 5% of total bacteria (not published). This is first report of complete genome analysis of the class *Fimbriimonadia* in the phylum *Armatimonadetes*.

## Materials and Methods

### Sequencing and assembly

*Fimbriimonas ginsengisoli* Gsoil 348^T^ isolated from ginseng field soils was cultured with diluted modified R2A medium [4]. Isolated genomic DNA from pure culture of strain Gsoil 348^T^ was sequenced using pyrosequencing approaches on Roche GS FLX platform, which produced a total of 323,309 reads with an average length of 363 bp. The coverage of entire genome was approximately 22-fold and after initial assembly using Newbler, and 49 large contigs with an average length of 107 kb were generated. Gap closure was accomplished by multiplex PCR and PCR sequencing after the PCR products were gel-extracted. The final genome sequence was assembled using Phrap (http://www.phrap.org). The low-quality sequences were resequenced by PCR sequencing using ABI 3730 and the final sequence accuracy was 99.994%. The genome sequence data were deposited in Genbank with the accession number CP007139.

### Gene prediction and annotation

Gene prediction was carried out on the strain Gsoil 348^T^ genome using GLIMMER, ZCURVE and RAST [PMID: 18261238] [7]–[9]. Functional annotation of CDSs was performed by searching against NCBI non-redundant protein database and KEGG protein database [10] using BLASTP, and combining the annotation result of RAST. Functional classification of predicted genes were performed based on KEGG Brite and RAST subsystem. The protein domains and COG [PMID: 10592175] assignment were predicted by RPS-BLAST against NCBI CDD library [PMID: 17135202].The metabolic pathways were constructed based on KEGG database. For enzymes lost in the Gsoil 348^T^ pathways, we download the reference protein sequences and search against predicted proteins to reconstruct the pathway. We also compared the annotation results with genomes of five typical microorganisms: Gram negative bacterium *Escherichina coli* DH10B, Gram positive bacterium *Bacillus subtilis* 168, actinobacteria in soil *Streptomyces coelicolor* A3(2), archaea *Methanosarcina acetivorans* C2A and slow growing bacterium *Mycobacterium tuberculosis* H37Rv.

### Phylogenetic analysis

Phylogenetic analysis of the strain Gsoil 348^T^ was based on the concatenated dataset of ribosomal protein sequences. As references, genome sequences of 103 strains (<97% 16S rRNA similarity) belonging to 19 main bacterial phyla which were available at the public database MBGD (Microbial Genome Database for Comparative Analysis) were selected. Ribosomal protein sequences were obtained via BLASTP searches rather than merely from annotated information. In total, 32 ribosomal protein sequences were selected (**Table S1 in File S1**). Concatenated protein sequences were aligned using MUSCLE [11], and then the conserved alignment blocks were extracted by the Gblocks program [12]. The phylogenetic tree was built using the maximum likelihood method implemented in PHYML [13] using following parameters: 1000 replications for bootstrap analysis, “JTT” for substitution model, “estimated” for proportion of invariable sites, “estimated” for gamma distribution parameters, “4” for the number of substitution categories, “yes” to optimize tree topology, and “BIONJ” for starting tree(s). Graphical representation and edition of the phylogenetic tree were performed with NJPlot [14].

## Results and Discussion

### General genome feature

**Figure 1:**
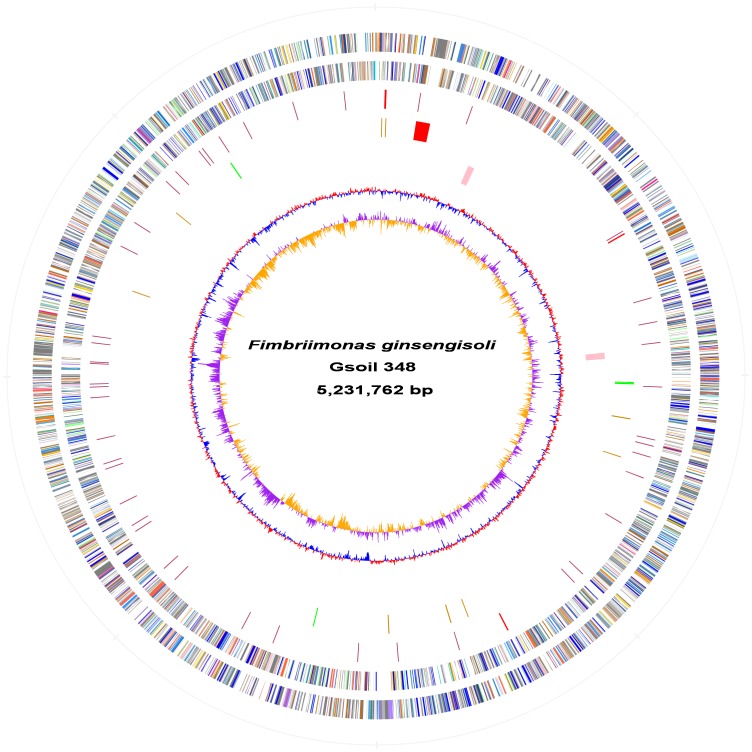
Genome Atalas of *Fimbriimonas ginsengisoli* Gsoil 348^T^. Moving inside, each concentric circle represents genomic data for strain Gsoil 348^T^. The outer two circles illustrate predicted coding sequences on the plus and minus strands, respectively, colored by functional categories according to COG classification. The 3rd circle displays rRNA (red) and tRNA (maroon). The 4^th^ circle shows loci of prophage (red), IS elements (orange) and the four horizontal transferring regions (green). The 5^th^ circle presents two putative gene clusters (pink). The 6^th^ circle represents mean centered G+C content of the genome (red-above mean, blue-below mean). The 7^th^ circle (innermost) represents GC skew (G-C)/(G+C) calculated using a 2 kb window in steps of 1 kb.

The complete genome of strain Gsoil 348^T^ contains a single circular chromosome of 5,231,762 bp with a G+C content of 60.8% (**Table 1**, **Figure 1**). In total, 4,820 ORFs were identified in the genome with an average length of 955 bp, occupying 88.0% of the genome. Among these ORFs, 3,000 (62.2%) genes could be classified into Clusters of Orthologous Groups (COG) families comprising 21 functional categories (**Table S2 in File S1**), and 1,282 domains were found in 3,072 genes (63.8%). Meanwhile, 3,553 genes (73.7%) had homology with known proteins, with an average identity of 39.6%. Combining with COG and Pfam analysis, a total of 3,704 ORFs (76.8%) were assigned putative biological functions, with the left 1,116 hypothetical proteins (23.2%) as strain Gsoil 348^T^ specific genes. Several enzyme activities had been checked in strain Gsoil 348^T^ [4], here we determined their corresponding genes, like alkaline phosphatase (OP10G_4235), beta-glucuronidase (OP10G_0307), 11 alpha-L-fucosidase genes, five alpha-galactosidase and 12 beta-galactosidase genes, etc.

**Table 1:** Genome Feature of Gsoil 348^T^ and other Reference strains.

	Strain Gsoil 348^T^	*E. coli*	*B. subtilis*	*S. coelicolor*	*M. acetivorans*	*M. tuberculosis*
Genome Size (bp)	5,231,762	4,686,137	4,215,606	8,667,507	5,751,492	4,411,532
GC content	61%	51%	44%	72%	42%	66%
Protein coding Genes	4,820	4,128	4,176	7,768	4,540	4,003
Coding region	88%	83%	87%	88%	73%	91%
tRNA	47	86	86	65	59	45
rRNA operons	1	7	10	6	3	1
COGs (ratio)	62.2%	85.1%	75.0%	70.1%	70.5%	72.8%

E. coli: Escherichia coli DH10B; B. subtilis: Bacillus subtilis 168; S. coelicolor: Streptomyces coelicolor A3(2); M. acetivorans: Methanosarcina acetivorans C2A; M. tuberculosis: Mycobacterium tuberculosis H37Rv.

The genome of strain Gsoil 348^T^ had no significant homology to known genomes. Indeed the best hit homologs of 3,585 proteins scattered in 1,143 species of 33 phyla, with *Firmicutes* (21.4%) and *Proteobacteria* (21.3%) most abundant followed by *Bacteroidetes* (7.4%), *Verrucomicrobia* (6.8%), *Chloroflexi* (6.6%), *Planctomycetes* (5.4%), *Actinobacteria* (5.2%), *Acidobacteria* (5.2%) and *Cyanobacteria* (4.6%) (**Table S3 in File S1**). However, the most matched strains were *Candidatus* Pedosphaera parvula Ellin514 (2.8%) in the phylum *Verrucomicrobia* and *Candidatus* Solibacter usitatus Ellin6076 (2.0%) in the phylum *Acidobacteria*.

The first base of the genome was chosen at the start codon of chromosomal replication initiator protein DnaA. Unlike other bacteria, strain Gsoil 348^T^ had no typical 16S-23S-5S ribosomal RNA operon. Instead, the 23S-5S rRNA operon was located 26 kb downstream of *dnaA*, and two 16S ribosomal RNA genes (100% similarity) located 875 kb and 2.2 Mb away, respectively (**Figure 1**). The split of rRNA genes were determined from host-dependent bacteria such as the genera *Buchnera*, *Mycoplasma*, *Helicobacter* and *Rickettsia*, and thermophilic bacteria *Thermus*; and all of these bacteria possess small genome size (<2 Mb) [15], [16]. Therefore, strain Gsoil 348^T^ is first relatively large genome with the split of rRNA genes.

A total of 46 tRNAs were revealed in the genome representing all 20 amino acids, with seven tRNA genes for strongly basic amino acids (Arg and Lys) and three tRNA genes for strongly acidic amino acids (Glu and Asp).

The proteome of strain Gsoil 348^T^ was composed of 1,530,250 amino acids with an average isolectric point of 6.52. The most abundant amino acids were Ala, Leu, Gly and Val, occupying 1/3 of the proteome, while Cys only accounted for 0.86%.

Bacteria usually have a codon preference for different amino acid. We studied the distribution of the 64 codons in the predicted genes, and the profile of each codon usage was compared with five reference strains (**Table S4 in File S1**). As a high GC content genome of strain Gsoil 348^T^, 75.7% of the codons have a G or C at the wobble position, versus 63.0% and 45.3% at the 1st and 2nd position of codon, respectively. *M. tuberculosis* (correlation coefficient: 89.2%) and *S. coelicolor* (88.0%) showed the most similar pattern with strain Gsoil 348^T^. However, unlike *S. coelicolor* and *M. tuberculosis*, which using TGA as favorite stop codon with a percentage of 77.7% and 54.6%, respectively; the three stop codons had an equal usage ratio for strain Gsoil 348^T^.

In addition, strain Gsoil 348^T^ encoded 1,282 kinds of domains, and 343 domains are common in the five reference genomes. Only 145 domains (11.3%) are unique for strain Gsoil 348^T^ relative to the five reference genomes (**Table S5 in File S1**). We also see 10 groups of duplicated genes in strain Gsoil 348^T^ genome including two duplicated peptidases (**Table S6 in File S1**).

Six kinds of COGs are detected to be abundant (p<0.001) in strain Gsoil 348^T^ than in five reference bacteria (**Table S7 in File S1**), including Ankyrin repeat COG0666 (from 12 genes: Arp), which is important for thermal nociception [17] and may make strain Gsoil 348^T^ to survive and adapt to its inhabits' environments.

### Prophage and IS element

Only 12 transposase genes were revealed in the genome, indicating that strain Gsoil 348^T^ lacked IS elements, or at least known IS elements. This was uncommon since its living niche was rich of bacteria and lateral gene transfer chances should be enormous. Meanwhile, we found a complete Type I restriction-modification system in the genome, perhaps inhibiting lateral gene transfer (**Table S8 in File S1**).

Four fragments were identified as horizontal transferring from other genomes (**Table S9 in File S1**). Noteworthy, a 1,587 bp fragment composed of two short-chain dehydrogenase/reductase SDR encoding genes (OP10G_1224 and OP10G_1225) showed 86% identity with *Mesorhizobium loti* MAFF303099 genome, indicating a possible close inhabiting environment.

Strain Gsoil 348^T^ contained one prophage (45.5 Kb, **Table S10 in File S1**). Through PCR detection, we confirmed that the prophage existed in genome with two stages: prophage inserted in genomic DNA and circled prophage not-inserted in genomic DNA; and there also detected genomic DNA not contain this prophage. The proteins predicted in the prophage genome included recombinase, primase, terminase, methylase and helicase. Through the sequence comparison, we found that the phage may be inserted to genomic DNA through site-specific recombination, and recombination position is completely matched. The prophage's remaining genome contains open reading frames (ORF) bordered by a duplicated core sequences of 20 nucleotides (GATTTCGAGAAATGGCAGGG). This core sequence is included in the site-specific recombinase gene. Therefore, during the insertion of this prophage, the end site of recombinase gene is from the sequence of prophage; during the deletion of this prophage, the end site of recombinase gene sequence is from the sequence of chromosomal genome (**Figure S1**). Core sequence including in the site-specific recombinase gene is not reported upto now, and its effect to the induction need further study.

### Replication origin and genetic information processing

The bacteria *oriC* region is frequently located within the *rnpA–rpmH–dnaA–dnaN–recF–gyrB* gene cluster [18]. However, we found no *rnpA* or *rpmH*, and the other genes scattered in the genome. *dnaN* located 5,018 kb in the genome, *recF* in 1,929 kb, and *gyrB* in 2,323 kb.

The *oriC* region is also characterized by DnaA box motifs of which the consensus sequence is 5′-TTATCCACA-3′, but we only found two DnaA box sequence at 4.34 Mb and 4.55 Mb position. Between *dnaA* and *dnaN* (210 kb region), we found one reverse complement sequence of DnaA box at 5.20 Mb, matching the replication origin of 5.13 Mb predicted by GCskew.

Although we found no features of a terminus of replication, a GC skew inversion was found at position 4,388,810, which could be considered the terminus of replication. Considering the “jagged” GC skew map (**Figure S2**), which was usually observed in archaea instead of in bacteria [19], we might speculate that the strain Gsoil 348^T^ genome underwent severe mutation forces in the soil environment.

We found only twelve DNA replication proteins in the genome (**Table S11 in File S1**) with another 34 genes involved in DNA repair systems (**Table S12 in File S1**). Three genes (*dna*B, *dna*G and ssb) were found to participate in DNA replication initiation but no genes involved in DNA replication termination were revealed in the genome. The central enzyme, the DNA polymerase III holoenzyme, comprises four genes that separately encode the subunits alpha (DnaE), beta (DnaN) and gamma/tau (DnaX), and delta' (HolB). In addition, five genes are involved in DNA elongation including two Rnase H genes, two DNA ligase genes and one DNA polymerase I gene (*polA*). The DNA repair systems in strain Gsoil 348^T^ were composed of base excision repair system, recombinational repair system, mismatch-repair system and nucleotide excision repair system. Two MutS genes and one MutL gene were revealed in the genome though the MutH gene was absent in the mismatch repair system.

Eighty-six genes are predicted to be involved in transcription (**Table S13 in File S1**), including four genes that encode the DNA-dependent RNA polymerase subunits (alpha, beta, beta' and omega), six genes encoding elongation and transcription termination factors (NusA, NusB, NusG, GreA and Rho), 40 genes (three *rpoD*, 30 *ropE* and one *fliA*) encoding sigma factors and another 36 transcription factors belonging to ten different families.

A total of 98 genes are involved in translation (**Table S14 in File S1**), including 42 ribosomal proteins, twelve translation initiation and elongation factors, and 33 genes involved in tRNA synthesis. And eleven genes involved in tRNA modification and another 40 genes participated in post translational modification (**Table S15 in File S1**).

### General metabolism

The genome of strain Gsoil 348^T^ had 983 enzyme-coding genes, among them 601 enzymes catalyzed 1,111 reactions in different metabolism pathways. Peptidases were mainly concentrated in metallo and serine peptidase (80% of peptidase). Compared with five reference bacteria, strain Gsoil 348^T^ had no significant difference in gene numbers involved in pathways and protein functional classification (**Table S16 and S17 in File S1**).

**Figure 2:**
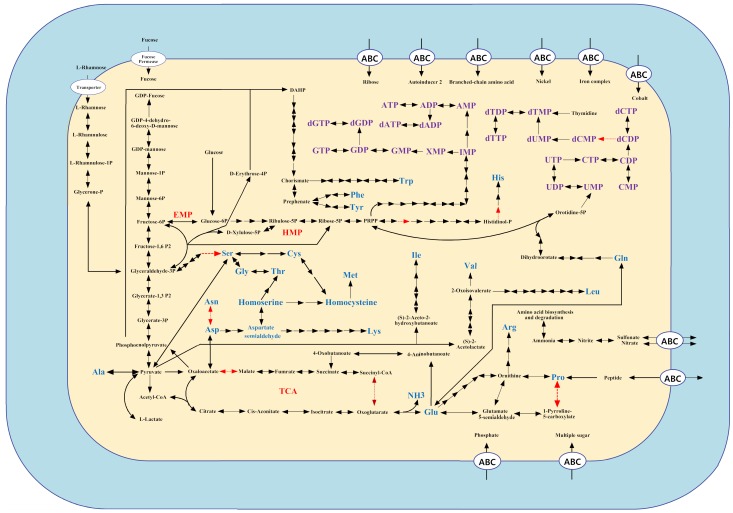
Schematic of metabolic pathways in strain Gsoil 348^T^. The black arrows represent enzymes existed in the genome, while the red arrows indicated that the enzyme was not encoded by the genome.

From the genome sequence, it is clear that strain Gsoil 348^T^ has the potential to synthesize fatty acids, purine and pyrimidine (**Figure 2**). The oxoglutarate was precursor of Glu, and would be transformed into Glu catalyzed by glutamate synthase (OP10G_3032). Glutamine would be produced by glutamine synthetase (OP10G_4181) from Glu. The oxaloacetate, which was produced from pyruvate, could be transformed into Asp catalyzed by L-aspartate aminotransferase (OP10G_2150 and OP10G_0324). Lys, Arg, Pro, Thr, Gly and Ser could be synthesized based on Asp. Ser could be produced from 3-phosphoglycerate, the mediate of gluconeogenesis, or from pyruvate by threonine dehydratase (OP10G_0216, OP10G_0723 and OP10G_4048). Cys and Met could be synthesized based on Ser; and Leu, Ile and Val could be synthesized based on pyruvate. Phe, Tyr and Trp could be synthesized from phosphoenolpyruvate (PEP) and D-Erythrose 4-phosphate. However, two genes (*hisE* and *hisB*) for His synthesis were not determined from the genome (**Figure 2**).

Bacteria may use gluconeogenesis to synthesize glucose from the intermediates of the tricarboxylic acid (TCA) cycle. A functional phosphoenolpyruvate carboxykinase (pckA) gene (OP10G_1890) was identified in strain Gsoil 348^T^, which catalyzed the synthesis of PEP from oxaloacetate, and endowed strain Gsoil 348^T^ with a complete gluconeogenesis pathway through the PpckA route. In addition, both pyruvate phosphate dikinase (Pps, OP10G_1859 and OP10G_3228) and phosphoenolpyruvate synthase (PpdK, OP10G_4434) were revealed in the genome, giving a hint that gluconeogenesis could occur from pyruvate.

However, malate could not be transformed into pyruvate, as no malate dehydrogenase was revealed from the genome. Glyoxylate cycle was uncompleted in the genome since enzymes transforming isocitrate to glyoxylate and glyoxylate to malate was not encoded.

### Metabolism of cofactors and vitamins

As a free living organism, Gsoil 348^T^ could synthesize several cofactors and vitamins (**Table S18 in File S1**). Strain Gsoil 348^T^ had enzymes synthesizing riboflavin (vitamin B2) from ribulose 5-phosphate, then could convert it into FMN and FAD through riboflavin kinase/FMN adenylyltransferase (OP10G_1358).

Nicotinate (vitamin B3), nicotinamide, NAD+ and NADP+ could be biosynthesized from Asp. Nicotinate ribonucleotide was first synthesized, then it will be transformed into nicotinate by nicotinate phosphoribosyltransferase (OP10G_4618), or converted into NAD+ catalyzed by Nicotinate-nucleotide adenylyltransferase (OP10G_3737) and NAD synthetase (OP10G_0664/4254). Both nicotinamide and NADP+ were produced from NAD+ (Table S17 in File S1).

Pantothenate (vitamin B5) could be produced by pantoate—beta-alanine ligase (OP10G_4346) from beta-alanine, which was converted from Asp by aspartate 1-decarboxylase (OP10G_3944). Then Dephospho-CoA could be produced from pantothenate through four enzyme catalyzed reactions, while dephospho-CoA kinase was absent in the genome, so CoA could not be produced. The way from pyruvate to pantothenate was not complete since 2-dehydropantoate 2-reductase [EC:1.1.1.169] was absent in strain Gsoil 348^T^.

Folate (vitamin B9) and Tetrahydrofolate (THF) could be synthesized from GTP and Chorismate (which would be converted into p-Aminobenzoate and feed in folate pathway),with a total of nine enzymes involved in this process. Heme could also be synthesized from glutamate with 11 enzymes catalyzing the process.

For pyridoxine (vitamin B6) metabolism, only two enzymes existed in genome of strain Gsoil 348^T^. One is pyridoxamine 5'-phosphate oxidase (OP10G_4084), which could produce pyridoxal 5-phosphate (PLP) from pyridoxamine phosphate (PMP) or pyridoxine phosphate (PNP). Another is Pyridoxine biosynthesis glutamine amidotransferase (OP10G_1471/2432), which could convert 5-phosphate ribose and 3-phosphate glyceraldehyde into PLP.

The enzymes involved in thiamine (vitamin B1) biosynthesis were completely absent in Strain Gsoil 348^T^, with only thiamin pyrophosphokinase (OP10G_4820) left which could convert thiamine into TPP, an important coenzyme. In addition, neither ubiquinone (Coenzyme Q10) nor biotin (vitamin B7) could be synthesized. Only two genes involved in vitamin K cycle was observed, one is *ubiE* which encoded ubiquinone/menaquinone biosynthesis methyltransferase (OP10G_1873), and another is NAD(P)H dehydrogenase (quinone) (OP10G_3171), which could transform Menaquinone/Phylloquinone to Menaquinol/Phylloquinol. This is in coincidence with the fact that the major respiratory quinones are menaquinones MK-11 and MK-10.

### Energy metabolism

Two pathways, Emden-Meyerhof-Parnas (EMP) pathway and hexose monophosphate (HMP) pathway, were complete to change glucose to pyruvate and change glucose-6P to NADPH and pentose, which would be the major energy generating pathways in strain Gsoil 348^T^. In fact, fructose-6P was produced from fructose by fructokinase (OP10G_0022),then participated in EMP pathway. For strain Gsoil 348^T^, TCA cycle was not complete with absence of the step from oxoglutarate to succinyl-CoA and malate to oxaloacetate in the genome. Thus the main role of the incomplete TCA was to convert pyruvate to necessary biosynthetic intermediates instead of producing energy. The main flow of pyruvate would be participating the TCA and involved in nitrogen metabolism through the oxoglutarate transforming into Glu.

When strain Gsoil 348^T^ is under aerobic growth conditions, ATP will be generated by oxidative phosphorylation from electron transport chains composed of NADH dehydrogenase (complex I), succinate dehydrogenase (complex II), cytochrome c oxidase, cytochrome bd complex and F-type ATPase except subunit c (**Table S19 in File S1**). The whole set of NADH-quinone oxidoreductase (complex I) were identified in strain Gsoil 348^T^, meanwhile SdhD of succinate dehydrogenase (complex II) was absent. In this case either NADH or succinate might be used as electron donors.

Although strain Gsoil 348^T^ cannot grow under anaerobic conditions [4], components of anaerobic phosphorylative electron transport chains are also present in strain Gsoil 348^T^, including genes transforming nitrate to ammonia (nitrate reductase and nitrite reductase) and sulfate to sulfite (**Table S20 in File S1**).

### Transporter systems, secretion systems and cell motility

Strain Gsoil 348^T^ showed an abundant amount of high-affinity and broad-specificity uptake systems, like the ATP-binding cassette (ABC) transporters. Strain Gsoil 348^T^ transporter system consists of 160 genes (**Table S21 in File S1**), which mainly constitute the ABC transporter system (137 genes). There was no phosphotransferase system (PTS) in the genome. The ABC transporter system included 39 ATP-binding proteins, 68 permease proteins and 28 substrate-binding proteins. We found a total of 29 set of complete ABC type transporters in the genome, mainly transporting sugars (17 sets), sulfate and antibiotics, with only one set having no definite substrate. Both fucose and rhamnose could be imported into cell, and rhamnose would be degraded into Glyceraldehyde 3-phosphate, then involved in EMP pathway. DppA (COG0747), detected in this genome, is the substrate binding component of the DppABCDF dipeptide transport system, which has been demonstrated to transport proline-containing dipeptides [20]. Dipeptide transporters of the DppA type have also been found to be capable of transporting heme and the heme precursor [21], indicating the potential scavenging of these iron-containing compounds from the surroundings. The gene encoding LivK (COG0683∶5), the periplasmic component of the high-affinity leucine-specific transport system, was also detected. We also found Mg(2+) transport P-type ATPase and Copper-translocating P-type ATPase in the genome in addition to seven genes of Ton and Tol transport systems.

Two component regulatory systems, consisting of a sensor protein kinase and a response regulator, are widespread among prokaryotes. In this genome, we found 18 genes encoding sensor kinases and 25 genes encoding response regulators (**Table S22 in File S1**). Unlike *B. subtilis* and *E. coli*, in which there are >30 copies of different two-component regulatory systems [22], strain Gsoil 348^T^ has only five complete pairs of sensor histidine kinases and response regulators, and a few isolated kinase and regulatory genes. This might indicate that the strain Gsoil 348^T^ inhabited in a relative simple environment, thus need not to develop abundant two-component systems to adapt complex living environment.

Strain Gsoil 348^T^ encodes 150 lipoproteins, 286 secreted proteins and 772 transmembrane proteins, indicating that 25.1% of encoded proteins are associated with the extracellular environment. Accordingly, four signal peptidase I genes and one lipoprotein signal peptidase gene were predicted in genome of strain Gsoil 348^T^. Meanwhile, 35 genes involved in secretion system were also revealed (**Table S23 in File S1**), including 21 genes in type II secretion system, five genes in type IV secretion system and one gene encoding TatC (OP10G_1975) involved in twin-arginine translocation system. The Sec (secretion) system was incomplete in the genome with only seven genes, with the absence of channel-forming proteins SecE and SecG in the genome. Consequently, proteins were assumed to be exported through signal-recognition particle (SRP) composed by Ffh (OP10G_4031) and FtsY (OP10G_1999). Only 286 secret proteins (5.9%) are predicted in strain Gsoil 348^T^, smaller than 819 secreted proteins (10.5%) from *S. coelicolor* which has ability to exploit nutrients in the soil [23].

Genes belonged to “cell motility and secretion” were obviously abundant in strain Gsoil 348^T^ (Table S2 in File S1), which is just coincidence with physical characteristics: attachment pilus, fimbriae. Extracellular pilus structures are common among bacteria, and have been implicated in diverse colonization functions [24]. There were 15 genes related with bacterial motility (**Table S24 in File S1**). Among them, eight genes encoded five kinds of pilus assembly proteins (PilB, C, D, Q, M), six genes encoded twitching motility protein (PilT) and one gene encoded ChpD which belonged to chemosensory pili system protein. Analysis using relevant COG and Pfam models shows 79 genes encoding pseudopilin PulG (COG2165) which is necessary for the type IV pilins' assembly [25], and 65 of 79 genes carry prokaryotic N-terminal methylation motif (pfam07963) which is often found at the N-terminal of pilins. The presence of multiple pilus genes is matched with the presence of fimbriae in strain Gsoil 348^T^ [4]. Because strain Gsoil 348^T^ does not exhibit motility, the pili are likely involved in attachment. The export systems of gram-negative bacteria can be divided into two main groups: the Sec-dependent general secretory pathway (including T2SS) and the Sec-independent secretion system [26]. Strain Gsoil 348^T^ participates on T2SS for extracellular transport depend on PulG cooperating with PulD (COG1450: 4), PulE, PulF, PulK, PulJ and PulO.

The genome of strain Gsoil 348^T^ had 19 genes encoding lipopolysaccharide (LPS) biosynthesis proteins (**Table S25 in File S1**), including one encoding WbpL (belonging to O-antigen), seven genes involved in lipid A formation, three genes participating in core oligosaccharide formation and seven genes participating in unusual sugar formation. It is matched with Gram negative of strain Gsoil 348^T^ with Gram staining.

Gram-positive bacteria usually has monoderm cell envelope (like *Actinobacteria* and *Firmicutes*) with necessary genes like sortase, while gram-negative bacteria has a diderm cell envelope (like *Proteobacteria*), with gene families specific for LPS production [27]. No sortase gene was identified in Gsoil 348^T^, and 14 out of 21 gene families enriched in archetypal diderm lineage was found (**Table S26 in File S1**). So we speculate that Gsoil 348^T^ had a diderm cell envelope.

Though seven glycosyltransferase proteins showed similarity with known EPS synthesis proteins, we did not reveal complete EPS cluster in the genome. But we can clearly show the capsule with photo microscope (**Figure S3**). In addition, a two component regulatory system, PhoB cooperated with PhoR, which is involved in exopolysaccharide (EPS) synthesis on phosphate limitation condition [28] is detected. It means may have different system for the production of capsules for the attachment to soil particle.

### Oxygen tolerance

Growth in the presence of oxygen would generate partially reduced reactive oxygen species, including hydrogen peroxide (H_2_O_2_), superoxide radicals (O_2_^–^) and hydroxyl radicals (HO), which would trigger DNA, lipids and proteins oxidation, causing lethal cellular damage [29]. Superoxide dismutases (SODs) and catalase/hydroperoxidases were the main defense mechanisms to scavenge superoxide radicals (O_2_^–^) and hydrogen peroxide (H_2_O_2_) in aerobic organisms, respectively, and thus prevent the formation of HO via the Fenton chemistry [30]. We identified SOD (OP10G_4577), catalase (OP10G_4330) and glutathione peroxidase (OP10G_3509) in strain Gsoil 348^T^ genome (**Table S27 in File S1**). Catalase was also determined by experiments in strain Gsoil 348^T^ [4].

Meanwhile, the disulfide-reducing pathway played an important role in oxygen tolerance, and we identified genes encoding thioredoxin system, a protein-disulfide isomerase (PDI) and two peptide methionine sulfoxide reductases in the genome. The thioredoxin system in strain Gsoil 348 included four thioredoxin reductases (COG0492, TrxB), nine thioredoxins (COG0526, TrxA) and nine thioredoxin domain containing proteins (Pfam00085). The PDI (OP10G_4063), containing four thioredoxin-like domains, catalyzed the formation and breakage of disulfide bonds between cysteine residues within proteins. Two peptide methionine sulfoxide reductases (COG0225) could reduce the oxidized form of methionine back to normal methionine [31].

Other possible proteins involved in oxygen tolerance included 13 AhpC/TSA family proteins (Pfam00578), which were related to alkyl hydroperoxide reductase (AhpC) and thiol specific antioxidant (TSA).

### Gene clusters

Two putative gene clusters were revealed in the genome (**Table S28 in File S1, Figure S4**). One cluster composed of 21 genes (OP10G_0338-OP10G_0358) produced antimicrobial compounds lantipeptide, another cluster composed of 24 genes (OP10G_1121-OP10G_1144) produced a terpene secondary metabolite.

### Effects to cell growth

Cell growth, or increase in cell mass, requires prodigious numbers of ribosomes, the molecular factories that carry out protein synthesis. In general, organisms that possess multiple rrn operons grow faster than those that possess one or two operons. It is reported that the low number of rRNA genes and transcriptional regulators, and the split of rRNA operons are related with low growth based on the comparison of free-living bacteria with host-dependent intracellular bacteria [15]. In the genome of strain Gsoil 348^T^, although there are two copes 16S rRNA genes and one 23S-5S rRNA operon, there are absent of complete 16S-23S-5S rRNA operon, showed split of rRNA genes as in first relatively large genome. The number of transcriptional regulators is 6.54 genes/Mb, similar with obligate intracellular bacteria (6.98±12.32 genes/Mb) but much lower than free-living bacteria (28.69±11.18 genes/Mb) [15].

Strain Gsoil 348^T^ could use peptone, casamino acids or yeast extract as sole carbon sources, however, could not use monosaccharide or disaccharide as sole carbon source [4]. It means that strain Gsoil 348^T^ needed amino acid substrates for cell growth. This phenomenon is also matched with the lack of His synthesis genes in the genome.

Strain Gsoil 348^T^ could not grow on high-nutrient media such as Luria-Bertani (LB) broth and nutrient agar (not published data), but grow on diluted R2A agar or broth. The main role of the incomplete TCA may limit the conversion of pyruvate to produce energy and might prevent glycolysis. The limited pyruvate usage pathway might prevent unblocked glycolysis, and it will increase the concentrate of this intermediate in the cells and cause toxicity to cells. It might partly explain why this strain only grows in low-nutrient medium such as diluted R2A [4].

### Evolutionary position of *Armatimonadetes* revealed by phylogenetic analysis

**Figure 3:**
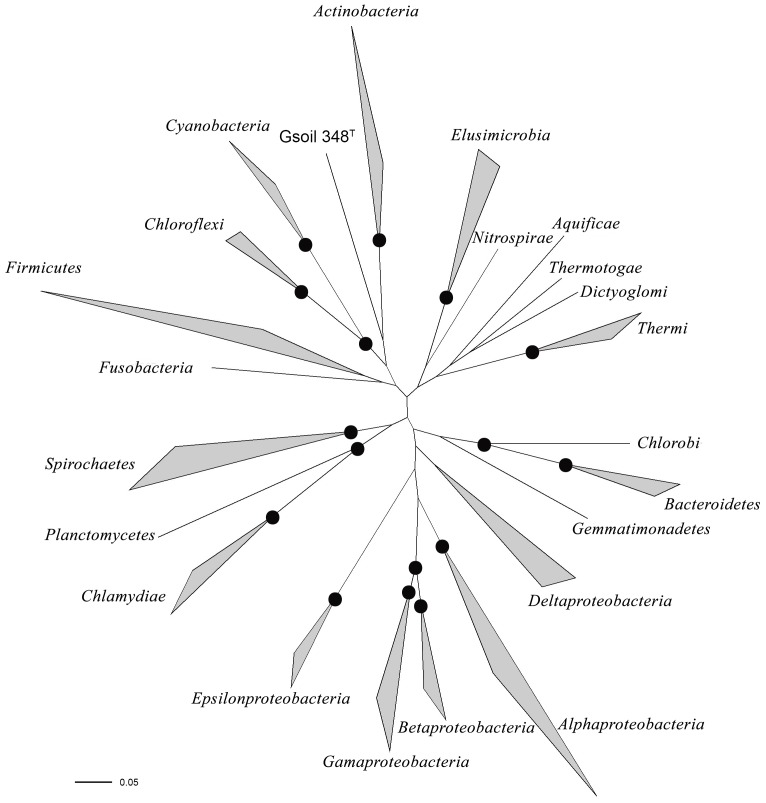
Unrooted phylogenetic tree based on 32 concatenated ribosomal protein sequences. It is showing the phylogenetic positioning of strain Gsoil 348^T^ within the Bacteria domain. Filled dark cycle at nodes represent 100% maximum likelihood-based bootstrap support. At the node of strain Gsoil 348^T^ and *Actinobacteria* showed represent 72% maximum likelihood-based bootstrap support.

Ribosomal proteins (RibP) are highly conserved in all bacteria species and could be used as the investigation of evolutionary positions [32]. A dataset of 32 concatenated RibP amino acid sequences were assembled from 104 representative bacterial genomes belong to 22 phyla (**Table S1 in File S1**). Phylogenetic analyses indicated that strain Gsoil 348^T^ represents a deeply branching lineage of sufficient divergence to be considered as a novel phylum, but also strongly correlated with phyla *Actinobacteria*, *Chloroflexi* and *Cyanobacteria* (**Figure 3**), which is congruent with the results of strain T49^T^ based on ribosomal protein [3] and based on rRNA genes [4], [6]. Therefore, phylum *Armatimonadetes* could be involved in superphylum *Terrabacteria* as suggested by some researchers [33], [34].

### Comparative analysis with *Chthonomonas calidirosea* T49^T^

During revision of this paper, we found an in press paper related with genome sequence of *Chthonomonas calidirosea* T49^T^ [35], another strain in the same phylum but in different class with strain Gsoil 348^T^. The *C. calidirosea* T49^T^ genome was only 3.4Mb, with GC content of 54.41% and encoding 2,811 protein encoding genes. Its genome was 1.8Mb smaller than strain Gsoil 348^T^. Genome alignment showed that the two genomes in the same phylum had almost no homologous regions (**Figure S5**). Protein similarity and synteny analysis between the two genomes also indicated that strain Gsoil 348^T^ had little similarity with *C. calidirosea* T49^T^ (**Figure S6**). Using protein identity >50% and alignment length coverage >50% as criteria, we identified only 413 ortholog clusters between the two genomes (**Table S29 in File S1**), leaving 84% (*C. calidirosea* T49^T^)-90% (strain Gsoil 348^T^) proteins as unique. It means that strain Gsoil 348^T^ much different with *C. calidirosea* T49^T^ in different class.

## Conclusion

In the metagenome or metatranscriptome analysis, taxonomic binning solely based on protein encoding genes currently generates an artificial bias against groups with few sequenced genomes, and correspondingly over-represents phyla with many sequenced genomes [36]. To overcome this severe problem, there are in urgent need of complete genome sequences of novel taxa. The genome of *F. ginsengisoli* Gsoil 348^T^ showed different characteristics with other already known bacteria, such as the split of rRNA genes, the GC skew inversion and incomplete TCA cycle. The first complete genome sequence of class *Fimbriimonadia* in phylum *Armatimonadetes*, will be the base of the analysis of metabolic pathways from genome and metagenome sequences, it will help us for the cultivation of “uncultured” microorganisms.

## Supporting Information

Figure S1 **The structure of prophage in the strain Gsoil 348^T^ genome.**
(TIF)

Figure S2 **GC skew map of the strain Gsoil 348^T^ genome.**
(TIF)

Figure S3 **Light micrograph of the strain Gsoil 348^T^ suspended in India ink.**
(TIF)

Figure S4 **Schematic of gene clusters in the strain Gsoil 348^T^ genome.** (A) Gene cluster synthesizing lantipeptide; (B) Gene cluster synthesizing terpene.
(TIF)

Figure S5 **Genome structure comparison between Gsoil 348^T^ and** ***Chthonomonas calidirosea*** **T49^T^**. The lines between the two genomes indicated homologous regions.
(TIF)

Figure S6 **Synteny between Gsoil 348^T^ and** ***Chthonomonas calidirosea*** **T49^T^**. x axis, position on Gsoil 348^T^ genome; y axis, position on *C. calidirosea* T49^T^ genome. Colors indicate protein similarity by BLAST score ratio according to the scale on the right.
(TIF)

File S1 **Contains the files: Table S1.** Thirty-two ribosomal protein sequences in 104 representative bacteria used for phylogenetic analysis. **Table S2.** COG classification in the strain Gsoil 348^T^ compared with five other species. **Table S3.** Distribution of best hit proteins in the genome. **Table S4.** 64 codons in the predicted genes in the strain Gsoil 348^T^ compared with five other species. **Table S5.** Unique domains in the genome. **Table S6**. Duplicated genes in the genome. **Table S7.** Abundance of functional genes in the strain Gsoil 348^T^ compared with five other species. **Table S8.** Complete type I restriction-modification system in the genome. **Table S9.** Lateral transfer of genes in the genome. **Table S10.** Prophage genes in the genome. **Table S11.** Genes involved in DNA replication in the genome. **Table S12.** Genes involved in DNA repair in the genome. **Table S13.** Genes involved in DNA transcription in the genome. **Table S14.** Genes involved in translation in the genome. **Table S15.** Genes involved in post modification in the genome. **Table S16.** The KEGG pathway in the strain Gsoil 348^T^ compared with five other species. **Table S17.** KEGG BRITE analysis in the strain Gsoil 348^T^ compared with five other species. **Table S18.** Genes involved in cofactor and vitamin biosynthesis in the genome. **Table S19.** Genes involved in oxidative phosphorylation in the genome. **Table S20.** Genes involved in nitrogen and sulfur metabolism in the genome. **Table S21**. Transport system in the genome. **Table S22.** Two-component systems in the genome. **Table S23.** Secretion system in the genome. **Table S24.** Genes involved in cell motility and T2SS in the genome. **Table S25.** Lipopolysaccharide biosynthesis proteins in the genome. **Table S26.** Gene number of each protein family enriched in monoderm or diderm lineage in the genome. **Table S27.** Genes involved in oxygen tolerance in the genome. **Table S28.** Putative gene clusters in the genome. **Table S29.** Ortholog analysis between strains Gsoil 348^T^ and *Chthonomonas calidirosea* T49^T^.
(XLSX)

## References

[1] Hugenholtz P, Pitulle C, Hershberger KL, Pace NR (1998) Novel division level bacterial diversity in a Yellowstone hot spring. J Bacteriol 180: 366–376. 9440526 10.1128/jb.180.2.366-376.1998 PMC106892

[2] Tamaki H, Tanaka Y, Matsuzawa H, Muramatsu M, Meng XY, et al (2011) *Armatimonas rosea* gen. nov., sp. nov., of a novel bacterial phylum, *Armatimonadetes* phyl. nov., formally called the candidate phylum OP10. Int J Syst Evol Microbiol 61: 1442–1447. 20622056 10.1099/ijs.0.025643-0

[3] Dunfield PF, Tamas I, Lee KC, Morgan XC, McDonald IR, et al (2012) Electing a candidate: a speculative history of the bacterial phylum OP10. Environ Microbiol 14: 3069–3080. 22497633 10.1111/j.1462-2920.2012.02742.x

[4] Im WT, Hu ZY, Kim KH, Rhee SK, Meng H, et al (2012) Description of *Fimbriimonas ginsengisoli* gen. nov., sp. nov. within the *Fimbriimonadia* class nov., of the phylum *Armatimonadetes* . Antonie Van Leeuwenhoek 102: 307–317. 22527625 10.1007/s10482-012-9739-6

[5] Lee KCY, Dunfield PF, Morgan XC, Crowe MA, Houghton KM, et al (2011) *Chthonomonas calidirosea* gen. nov., sp nov., an aerobic, pigmented, thermophilic micro-organism of a novel bacterial class, *Chthonomonadetes* classis nov., of the newly described phylum *Armatimonadetes* originally designated candidate division OP10. Int J Syst Evol Microbiol 61: 2482–2490. 21097641 10.1099/ijs.0.027235-0

[6] Portillo MC, Gonzalez JM (2009) Members of the Candidate Division OP10 are spread in a variety of environments. World J Microbiol Biotechnol 25: 347–353.

[7] Salzberg SL, Delcher AL, Kasif S, White O (1998) Microbial gene identification using interpolated Markov models. Nucleic Acids Res 26: 544–548. 9421513 10.1093/nar/26.2.544 PMC147303

[8] Guo FB, Ou HY, Zhang CT (2003) ZCURVE: a new system for recognizing protein-coding genes in bacterial and archaeal genomes. Nucleic Acids Res 31: 1780–1789. 12626720 10.1093/nar/gkg254 PMC152858

[9] Aziz RK, Bartels D, Best AA, DeJongh M, Disz T, et al (2008) The RAST Server: rapid annotations using subsystems technology. BMC Genomics 9: 75. 18261238 10.1186/1471-2164-9-75 PMC2265698

[10] Kanehisa M, Goto S, Kawashima S, Okuno Y, Hattori M (2004) The KEGG resource for deciphering the genome. Nucleic Acids Res 32: D277–280. 14681412 10.1093/nar/gkh063 PMC308797

[11] Edgar RC (2004) MUSCLE: multiple sequence alignment with high accuracy and high throughput. Nucleic Acids Res 32: 1792–1797. 15034147 10.1093/nar/gkh340 PMC390337

[12] Castresana J (2000) Selection of conserved blocks from multiple alignments for their use in phylogenetic analysis. Mol Biol Evol 17: 540–552. 10742046 10.1093/oxfordjournals.molbev.a026334

[13] Guindon S, Gascuel O (2003) A simple, fast, and accurate algorithm to estimate large phylogenies by maximum likelihood. Syst Biol 52: 696–704. 14530136 10.1080/10635150390235520

[14] Perriere G, Gouy M (1996) WWW-query: an on-line retrieval system for biological sequence banks. Biochimie 78: 364–369. 8905155 10.1016/0300-9084(96)84768-7

[15] Merhej V, Royer-Carenzi M, Pontarotti P, Raoult D (2009) Massive comparative genomic analysis reveals convergent evolution of specialized bacteria. Biol Direct 4: 13. 19361336 10.1186/1745-6150-4-13 PMC2688493

[16] Boyer SL, Flechtner VR, Johansen JR (2001) Is the 16S-23S rRNA internal transcribed spacer region a good tool for use in molecular systematics and population genetics? A case study in cyanobacteria. Mol Biol Evol 18: 1057–1069. 11371594 10.1093/oxfordjournals.molbev.a003877

[17] Hwang RY, Stearns NA, Tracey WD (2012) The ankyrin repeat domain of the TRPA protein painless is important for thermal nociception but not mechanical nociception. PLoS One 7: e30090. 22295071 10.1371/journal.pone.0030090 PMC3266251

[18] Mackiewicz P, Zakrzewska-Czerwinska J, Zawilak A, Dudek MR, Cebrat S (2004) Where does bacterial replication start? Rules for predicting the *oriC* region. Nucleic Acids Res 32: 3781–3791. 15258248 10.1093/nar/gkh699 PMC506792

[19] Grigoriev A (1998) Analyzing genomes with cumulative skew diagrams. Nucleic Acids Res 26: 2286–2290. 9580676 10.1093/nar/26.10.2286 PMC147580

[20] Olson ER, Dunyak DS, Jurss LM, Poorman RA (1991) Identification and characterization of *dppA*, an *Escherichia coli* gene encoding a periplasmic dipeptide transport protein. J Bacteriol 173: 234–244. 1702779 10.1128/jb.173.1.234-244.1991 PMC207180

[21] Letoffe S, Delepelaire P, Wandersman C (2006) The housekeeping dipeptide permease is the *Escherichia coli* heme transporter and functions with two optional peptide binding proteins. Proc Natl Acad Sci U S A 103: 12891–12896. 16905647 10.1073/pnas.0605440103 PMC1568943

[22] Kunst F, Ogasawara N, Moszer I, Albertini AM, Alloni G, et al (1997) The complete genome sequence of the gram-positive bacterium *Bacillus subtilis* . Nature 390: 249–256. 9384377 10.1038/36786

[23] Bentley SD, Chater KF, Cerdeno-Tarraga AM, Challis GL, Thomson NR, et al (2002) Complete genome sequence of the model actinomycete *Streptomyces coelicolor* A3(2). Nature 417: 141–147. 12000953 10.1038/417141a

[24] Sauer FG, Mulvey MA, Schilling JD, Martinez JJ, Hultgren SJ (2000) Bacterial pili: molecular mechanisms of pathogenesis. Curr Opin Microbiol 3: 65–72. 10679419 10.1016/s1369-5274(99)00053-3

[25] Kohler R, Schafer K, Muller S, Vignon G, Diederichs K, et al (2004) Structure and assembly of the pseudopilin PulG. Mol Microbiol 54: 647–664. 15491357 10.1111/j.1365-2958.2004.04307.x

[26] Stathopoulos C, Hendrixson DR, Thanassi DG, Hultgren SJ, St Geme JW III, et al (2000) Secretion of virulence determinants by the general secretory pathway in gram-negative pathogens: an evolving story. Microbes Infect 2: 1061–1072. 10967286 10.1016/s1286-4579(00)01260-0

[27] Albertsen M, Hugenholtz P, Skarshewski A, Nielsen KL, Tyson GW, et al (2013) Genome sequences of rare, uncultured bacteria obtained by differential coverage binning of multiple metagenomes. Nat Biotechnol 31: 533–538. 23707974 10.1038/nbt.2579

[28] Janczarek M (2011) Environmental signals and regulatory pathways that influence exopolysaccharide production in rhizobia. Int J Mol Sci 12: 7898–7933. 22174640 10.3390/ijms12117898 PMC3233446

[29] Thibessard A, Borges F, Fernandez A, Gintz B, Decaris B, et al (2004) Identification of *Streptococcus thermophilus* CNRZ368 genes involved in defense against superoxide stress. Appl Environ Microbiol 70: 2220–2229. 15066816 10.1128/AEM.70.4.2220-2229.2004 PMC383142

[30] Bruno-Barcena JM, Andrus JM, Libby SL, Klaenhammer TR, Hassan HM (2004) Expression of a heterologous manganese superoxide dismutase gene in intestinal lactobacilli provides protection against hydrogen peroxide toxicity. Appl Environ Microbiol 70: 4702–4710. 15294805 10.1128/AEM.70.8.4702-4710.2004 PMC492360

[31] Cabreiro F, Picot CR, Friguet B, Petropoulos I (2006) Methionine sulfoxide reductases: relevance to aging and protection against oxidative stress. Ann N Y Acad Sci 1067: 37–44. 16803968 10.1196/annals.1354.006

[32] Strous M, Pelletier E, Mangenot S, Rattei T, Lehner A, et al (2006) Deciphering the evolution and metabolism of an anammox bacterium from a community genome. Nature 440: 790–794. 16598256 10.1038/nature04647

[33] Rinke C, Schwientek P, Sczyrba A, Ivanova NN, Anderson IJ, et al (2013) Insights into the phylogeny and coding potential of microbial dark matter. Nature 499: 431–437. 23851394 10.1038/nature12352

[34] Yutin N, Puigbo P, Koonin EV, Wolf YI (2012) Phylogenomics of prokaryotic ribosomal proteins. PLoS One 7: e36972. 22615861 10.1371/journal.pone.0036972 PMC3353972

[35] Lee KC-Y, Morgan XC, Dunfield PF, Tamas I, McDonald IR, et al. (2014) Genomic analysis of *Chthonomonas calidirosea*, the first sequenced isolate of the phylum *Armatimonadetes*. ISME J doi: 10.1038/ismej.2013.251 [Epub ahead of print]. 10.1038/ismej.2013.251 PMC4069393 24477196

[36] Urich T, Lanzen A, Qi J, Huson DH, Schleper C, et al (2008) Simultaneous assessment of soil microbial community structure and function through analysis of the meta-transcriptome. PLoS One 3: e2527. 18575584 10.1371/journal.pone.0002527 PMC2424134

