# Supplementary figures and images for: The First Complete Genome Sequence of the Class Fimbriimonadia in the Phylum Armatimonadetes
Source: PLoS One. 2014 Jun 26;9(6):e100794. doi: 10.1371/journal.pone.0100794 (PMC4072686; doi:10.1371/journal.pone.0100794)

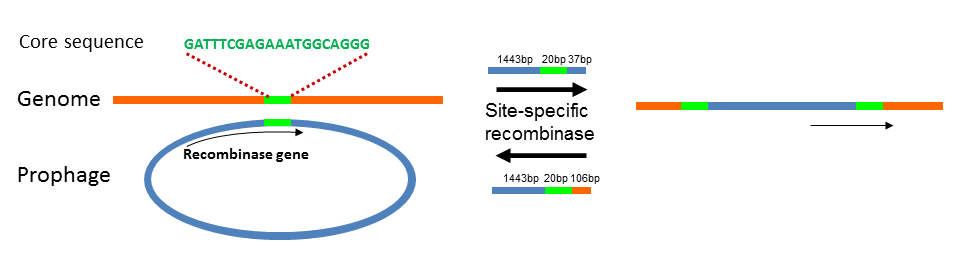

Supplement: Figure S1 — The structure of prophage in the strain Gsoil 348T genome. (TIF) [file pone.0100794.s001.tif]

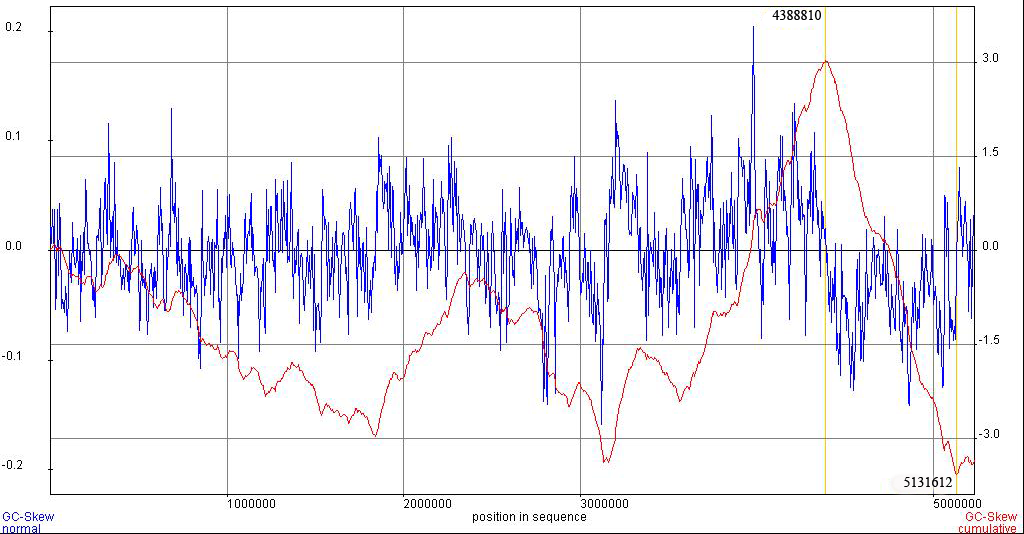

Supplement: Figure S2 — GC skew map of the strain Gsoil 348T genome. (TIF) [file pone.0100794.s002.tif]

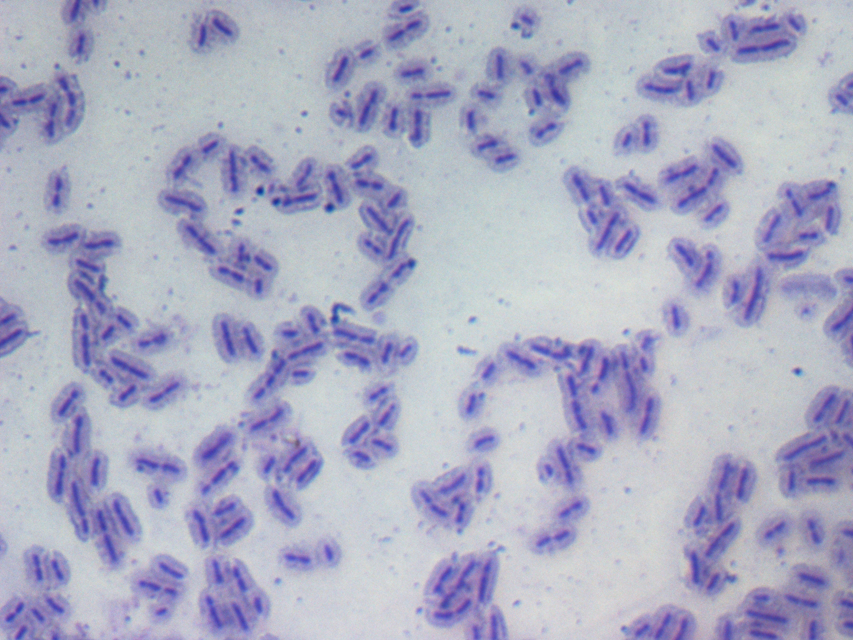

Supplement: Figure S3 — Light micrograph of the strain Gsoil 348T suspended in India ink. (TIF) [file pone.0100794.s003.tif]

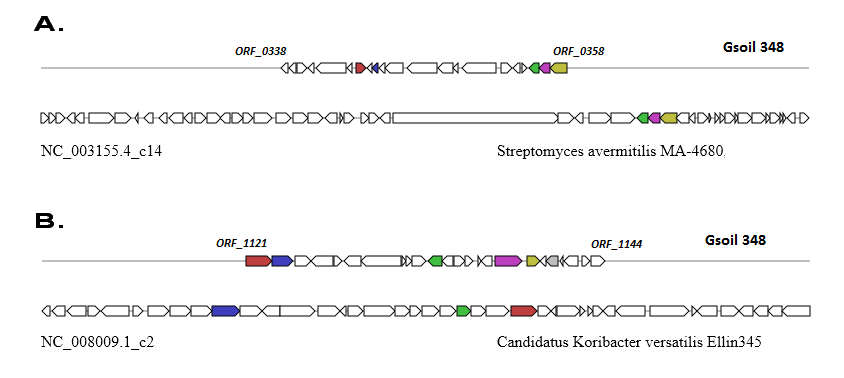

Supplement: Figure S4 — Schematic of gene clusters in the strain Gsoil 348T genome. (A) Gene cluster synthesizing lantipeptide; (B) Gene cluster synthesizing terpene. (TIF) [file pone.0100794.s004.tif]

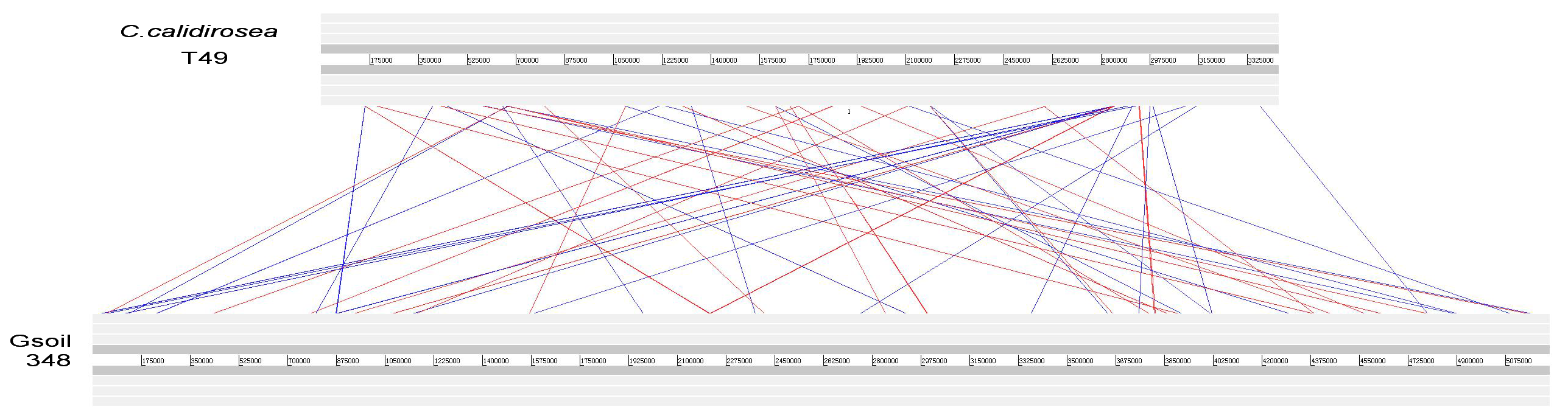

Supplement: Figure S5 — Genome structure comparison between Gsoil 348T and Chthonomonas calidirosea T49T. The lines between the two genomes indicated homologous regions. (TIF) [file pone.0100794.s005.tif]

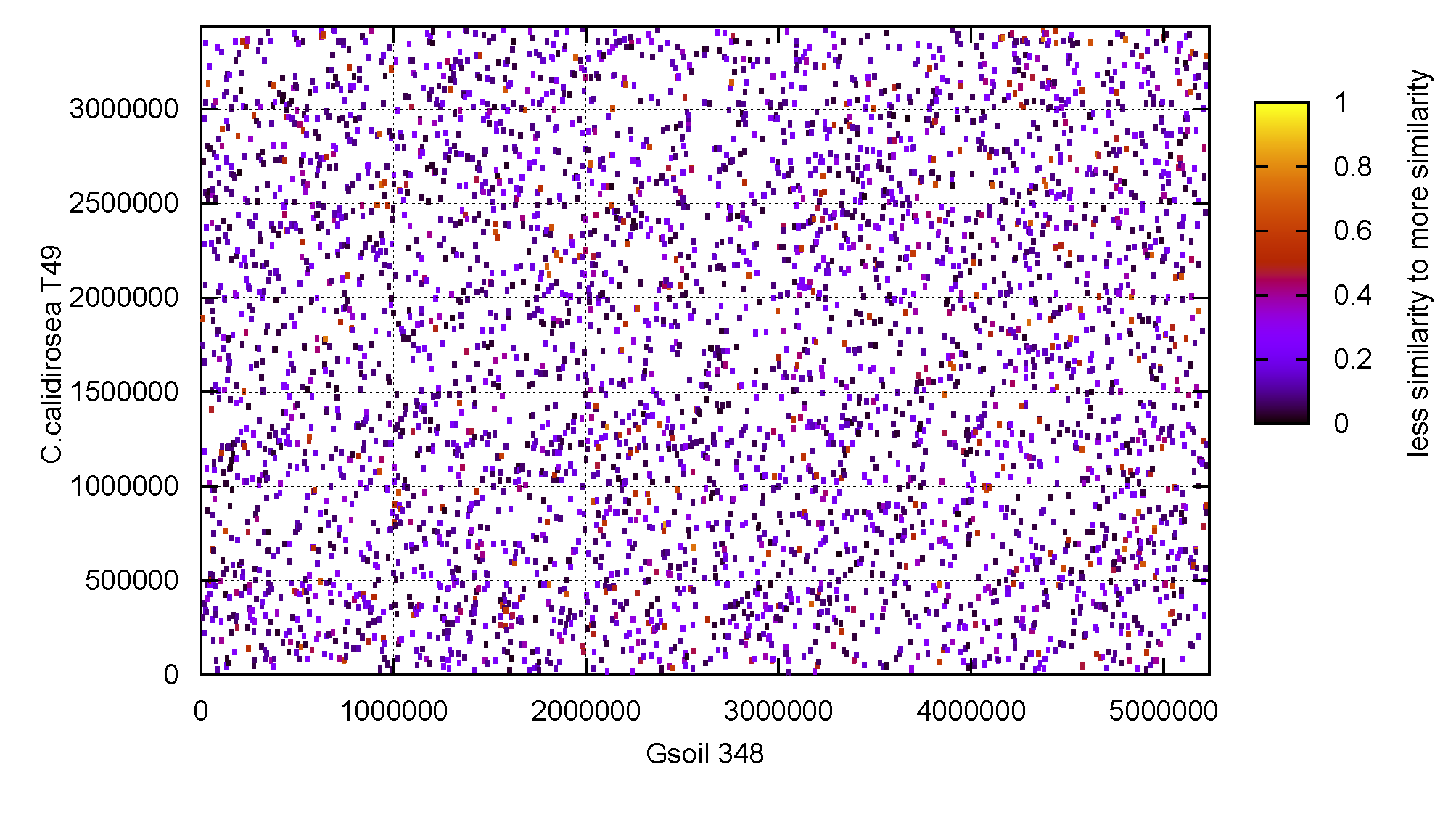

Supplement: Figure S6 — Synteny between Gsoil 348T and Chthonomonas calidirosea T49T. x axis, position on Gsoil 348T genome; y axis, position on C. calidirosea T49T genome. Colors indicate protein similarity by BLAST score ratio according to the scale on the right. (TIF) [file pone.0100794.s006.tif]
